# Static bending analysis of pressurized cylindrical shell made of graphene origami auxetic metamaterials based on higher-order shear deformation theory

**DOI:** 10.1016/j.heliyon.2024.e36319

**Published:** 2024-08-14

**Authors:** Mohammad Hossein Samadzadeh, Mohammad Arefi, Abbas Loghman

**Affiliations:** Department of Solid Mechanics, Faculty of Mechanical Engineering, University of Kashan, Kashan, Iran

**Keywords:** Cylindrical shell, Graphene origami, Internal and external pressures, Higher-order shear theory and normal deformation theory, Static bending

## Abstract

Static bending responses of a pressurized composite cylindrical shell made of a copper matrix reinforced with functionally graded graphene origami are studied in this paper. The kinematic relations are extended based on a new higher-order shear and normal deformation theory in the axisymmetric framework. The constitutive relations are extended for the composite cylindrical shell where the effective modulus of elasticity, Poisson's ratio, thermal expansion coefficient and density are estimated using the Halpin-Tsai micromechanical model and the rule of mixture. Some modified coefficients are employed for correction of the mentioned material properties in terms of the volume fraction and the folding degree of graphene origami, characteristics of copper and graphene nanoplatelets and thermal loads. The principle of virtual work is used to derive governing equations through computation of strain energy and external work. The static bending results including radial and axial displacements, circumferential strain and stress are presented along the longitudinal and radial directions in terms of volume fraction, folding degree and distribution of graphene origami. The results show an increase in radial displacement and circumferential strain with an increase in folding degree and a decrease in volume fraction of graphene origami. The main novelty of this work is investigating the effect of foldability parameter and various distribution of graphene origami on static results of short cylindrical shell.

## 1 Introduction

Shell structures are extensively used in airplane structures, pressure vessels, chemical reactors and aerospace structures and vehicles. The cylindrical pressure vessels are most applicable shell structures in various technical situations. The auxetic metamaterials are currently introduced as a significant class of metamaterials with emerging characteristics applicable in innovative areas. Graphene origami made of folded graphene sheets using hydrogenerations are introduced and used in engineering structures and reinforced materials with tunable material properties. The negative Poisson's ratio as well as thermal expansion coefficient is introduced as main novelty of these materials. This paper introduces application of graphene origami auxetic metamaterials as a reinforcement in a cylindrical pressure vessel. After this introduction, a bending analysis is performed using a higher-order shear deformable model. A literature review is organized to show highlights of recent and more related works in the context of graphene origami and pressure vessels.

Rui et al. [1] extended a novel multi-functional model for thermodynamic analysis of a novel structure. Analysis of mechanical behavior of advanced structures was performed using optimization methods for identifying the crack modes [2]. The new composite materials and structures have been developed in the recent works using new reinforcement and composition [3,4]. Effect of thermal load was studied on the deformation and stress analyses of laminated composite plates by Guo et al. [5]. Effect of porous core material as well as nanofillers in carbon nanotube configuration was studied on the nonlinear responses of sandwich plate by Arefi and Rahimi [6]. Application of neural network and various optimization methods was extended for analysis of novel composite materials and advanced compositions [[7], [8], [9]]. Mohammadi et al. [10] presented an analysis on the thermoelastic behavior of constrained nanocomposite reinforced cylindrical shell.

Khalili et al. [11] employed a new three-dimensional theory using higher-order kinematic terms for free vibration responses of cylindrical shell. The results were compared with those results of thick cylindrical shells based on various shear deformation theories. Nwoji et al. [12] extended the higher-order shear deformation theory proposed by Reddy and Liew for static bending responses of isotropic circular cylindrical shells based on principle of virtual work. Ghannad et al. [13] investigated effect of clamped-clamped boundary conditions on the bening responses of axisymmetric homogeneous thick-walled cylindrical pressure vessels subjected to thermal and mechanical loads based on a lower-order shear deformation theory. The derived governing equations using principle of virtual work were solved using perturbation technique for a variable thickness cylindrical shell. Governing equations of motion were derived for analysis of a functionally graded cylindrical microshell based on extended shear deformation theory by Tadi beni et al. [14]. The size dependency was accounted based on modified couple stress theory in which micro length scale parameter was included in the constitutive relations. Effect of various characteristics of carbon nanotubes was studied on the electro-elastic analysis of pressurized cylindrical vessel sandwiched by two inner and outer piezoelectric rings based on higher-order shear deformation theory. The electro-elastic bending results were presented in terms of volume fractions and distributions of carbon nanotubes.

Arefi et al. [15,16] studied free vibration analysis of a functionally graded cylindrical shell sandwiched by two inner and outer piezoelectric rings. The kinematic relations were developed using von-Karman relations and shear deformation theory. Breslavsky et al. [17] studied static and dynamic responses of an hyperelastic cylindrical pressure vessel subjected to two types of compressive distributed loads. Buckling analysis of cylindrical pressurized shell was studied by Takeki and Takahiro [18] based on a numerical method and considering surface tension. Shear deformation theory was used for free vibration analysis of functionally graded orthotropic cylindrical shell by Sofiyev [19]. The kinematic relations were developed based on von-Karman nonlinear relations. The results were explored to discuss the effect of various profiles of functionalities, nonlinear strain and geometric parameters on the nonlinear vibration responses. A two dimensional thermal analysis was performed by Kushnir et al. [20] for elastic stress and strain analyses of functionally graded cylindrical pressure vessels with finite length. Thermoelastic responses were presented in terms of significant parameters along the coordinate system. The heat transfer equation was solves using Fourier heat transfer equation. The transient heat transfer equation was solved using Laplace and Fourier transformations. The thermoelastic responses were derived for simply-supported boundary conditions. The numerical results were presented along the radial and circumferential directions. The effect of various types of boundary conditions was studied on the free vibration responses of functionally graded cylindrical shell by Haddadpour et al. [21]. The gradation of material properties was assumed along the thickness direction. The thermal analysis was performed using the various temperatures at inner and outer surfaces. The governing equations were derived based on von-Karman nonlinear strain assumption.

Malekzadeh and Heydarpour [22] employed first-order shear deformable model for dynamic responses of rotating cylindrical shell subjected to thermal environement made of graded materials along the radial direction. Gillman et al. [23] extended a comprehensive work on the optimization of origami structures using Miora-ori materials based on truss model. There are some important works on the graphene-based structures [24,25]. Effect of initial voltage and a nonlinear analysis was studied on the analysis of sandwich piezoelectric beam by Azrar et al. [26] using some assumptions and simplifications for development of dynamic responses. Molecular dynamic was employed to estimate emerging properties of metamaterials by Wei et al. [27]. Main improvement of various material properties was described through folding the graphene into a three dimensional nanostructure. Arefi [28] presented a comprehensive multi-field formulation for analysis of a hybrid structure using curvilinear coordinate system and tensor analysis. Effect of nano and micro scale associated with size depend theory was accounted for static/dynamic analysis of composite sandwich structures [29,30]. A new shear and normal deformable model with accounting thickness stretching was developed for analysis microbeam based on a novel theory including three micro scale lengths [31]. Effect of finite length was studied on the static analysis of cylindrical shell composed of variable material properties using eigenvalue-eigenvector method [[32], [33], [34]]. Arefi and Rahimi [35] developed a new model for accounting effect of axial boundary conditions on the thermo electro elastic analysis of a FG cylindrical shell. Effect of higher-order shear deformable model based on thickness stretching concept was studied on the analysis of sandwich shell by Zhnag et al. [36]. Linear free vibration responses of graphene nanoplatelets reinforced beam with tunable material properties were studied by Arefi et al. [37]. The important discussion on the graphene origami and its effective material properties was included in this work. Bending and free vibration responses of sandwich cylindrical panel was studied using a refined kinematic models [38]. Some basic relations for elastic analysis of the structures can be observed in references [[39], [40], [41], [42]].

Dynamic-based formulation and analysis of novel nanocomposite reinforced structures was studied in the recent works [[43], [44], [45]]. Tien et al. studied effect of nonlinear strain components on the dynamic and chaos of reinforced plate subjected to multi field loading [43]. Effect of porous composition and nanocomposite reinforcement was studied on the thermally-induced vibration of sandwich shell [44]. Ha et al. [45] studied effect of honeycomb structure and its characteristics on the chaos analysis of cylindrical panels. Ha et al. [46] studied nonlinear dynamic analysis of a variable thickness plate made of porous materials. The solution procedure was obtained using Galerkin's method and the numerical results were obtained using finite element approach. Dzung et al. [47] studied effect of variable edges on the nonlinear dynamic responses of the reinforced nanocomposite plates including cracks. Zhou et al. [48] studied the impact of graphene nanoplatelets and porosity properties of reinforced spherical caps on the buckling responses of the composite structure. Shear mode of the buckling phenomenon was analyzed for a functionally graded porous sector annular plate reinforced with graphene nanoplatelets [49]. Babaei et al. [50] studied three-dimensional vibrational results of cylindrical panel reinforced with graphene. Mollaei et al. [51] studied torsional buckling results of a laminated cylindrical panel reinforced with graphene reinforcement. Effect of thermal loads was studied on the thermal stress distributions of rotating graphene nanoplatelet reinforced truncated conical shell [52].

Graphene origami is known as three-dimensional nanocomposite materials. Effect of various dispersion of nanotube reinforcement was studied on the thermoelastic responses of a pressurized cylindrical vessel using a shear deformable model [53]. Application of novel materials such as graphene was explained for manufacturing the lithium ion battery by Meng et al. [54]. Wang et al. [55] developed a new programmable method for estimation of the effective material properties of nanocomposite reinforced structure. Graphene origami as an auxetic material can be used in the environement with multi-fied loading conditions. Lin et al. developed a dynamic-based model for analysis of loaded dock subjected to water pressure [56]. The new mathematical methods are needed for solution of the governing equations of motion in the new physical problems. There are some examples for application of new mathematical models for analysis of satellite trajectory in the literature [57,58]. Impact of a porous core as well as graphene origami reinforcement was studied on the higher-order static bending responses of cylindrical shell by Adab et al. [59]. An investigation on the large negative value of Poisson's ratio on the mechanical behavior of foldable structure was performed by Moradweysi et al. [60]. The new development in the material science for propose of novel reinforcement and advanced compositions has been suggested in detail by researchers [[61], [62], [63], [64]]. Mohammadzadeh et al. [65,66] developed new fuzzy neural networks for optimization and sensitivity analysis of high dimensional issues and nonlinear control of systems. Application of various neural network methods was described in the recent works [[67], [68], [69]]. Application of hybrid materials and structures such as nanocomposite structure were studied by recent researchers [[70], [71], [72], [73], [74]]. Material scientists and engineers suggested novel materials and structures with novel properties. There are some reports for investigating the effective material properties in therms of significant parameters [[75], [76], [77], [78], [79]]. The micromechanical behavior and material science characteristics of novel materials and structures was studied by Zhao et al. [80], Xu et al. [81] and Zhang et al. [82]. The effect of some mechanical operations such as welding and rolling was studied on the stress distribution of novel structures by Zhu et al. [83], Qian et al. [84] and Deng et al. [85]. The cross sectional distortion phenomenon was explained for a variable radius tube by Wang et al. [86], Wang et al. [87] and Li et al. [88]. There are some applications of material with irregular and expevted properties such as developed by Wang et al. [89], Shen et al. [90], Liu et al. [91], Gao et al. [92] and Meng et al. [93].

We organized a review study on the graphene origami metamaterials and cylindrical pressure vessels. The review was indicated that the bending responses of grapehen origami reinforced cylindrical pressure vessels is necessary for design of the novel reinforced structures. In this paper, we suggest application of graphene origami auxetic metamaterials as a tunable reinforcement for improvement of the mechanical behavior of pressure vessels subjected to mechanical loads. A higher-order shear and normal deformation theory is employed to simulate kinematic deformation and the constitutive relations are extended in the cylindrical coordinate system. The results will be presented in terms of adjustable parameters of the graphene origami along the radial and axial directions. The main novelty of this paper is accounting effect of foldable structure such as foldability parameter, graphene origami content and thermal loads on the static results of the reinforced composite shell.

## 2 Formulation

The bending differential equations of a pressurized reinforced cylindrical shell are derived in this section. The cylindrical shell is reinforced with graphene origami auxetic metamaterials. The effective material properties of the mentioned materials including modulus of elasticity, Poisson's ratio, density and thermal expansion coefficient are derived for a copper matrix reinforced with grapehen origami are derived as follows [24,94,95]:

(1) $$E_{C}=\frac{1+\xi \eta V_{Gr}}{1-\eta V_{Gr}}E_{Cu}\times f_{E}\left(H_{Gr},V_{Gr},T\right)$$

(2) $$\nu_{c}=\left(\nu_{Gr}V_{Gr}+\nu_{Cu}\;V_{Cu}\right)\times f_{v}\left(H_{Gr},V_{Gr},T\right)$$

(3) $$\alpha_{c}=\left(\alpha_{Gr}V_{Gr}+\alpha_{Cu}V_{Cu}\right)\times f_{\alpha }\left(V_{Gr},T\right)$$

(4) $$\rho_{c}=\left(\rho_{Gr}V_{Gr}+\rho_{Cu}V_{Cu}\right)\times f_{\rho }\left(V_{Gr},T\right)$$

In which E_c_، ν_c_، α_c_، ρ_c_ are effective density, effective modulus of elasticity, effective Poisson's ratio, and effective thermal expansion coefficient in Eqs. (1), (2), (3), (4), respectively. In addition, V_Gr_ η, ξ are volume fraction, material and size coefficients respectively that are expressed as follows [94,96]:

(5) $$V_{Gr}=\frac{\rho_{Gr}W_{Gr}}{\rho_{Gr}W_{Gr}+\rho_{Gr}\left(1-W_{Gr}\right)}$$

(6) $$V_{Cu}=1-V_{Gr}$$

(7) $$\xi =2\frac{l_{Gr}}{t_{Gr}}$$

(8) $$\eta =\frac{\frac{E_{Gr}}{E_{Cu}}-1}{\frac{E_{Gr}}{E_{Cu}}+\xi }$$

In which W_Gr_, l_Gr_, t_Gr_ are mass fraction, length and thickness of graphene origami, respectively based on Eqs. (5), (6), (7), (8). Furthermore, the subscripts Gr, Cu are used for graphene origami and copper, respectively.

The modifier coefficients (Eqs. (9), (10), (11), (12)) are derived for modification of modulus of elasticity, Poisson's ratio, thermal expansion coefficient and density in terms of volume fraction $V_{Gr}$ and folding degree $H_{Gr}$ of graphene origami and thermal loading T [94].

(9) fE(HGr,VGr,T)=1.11−1.22VGr−0.134(TT0)+0.559VGr(TT0)−5.5VGrHGr+38HGrVGr2−20.6HGr2VGr2

(10) fv(HGr,VGr,T)=1.01−1.43VGr+0.165(TT0)+0.559VGr(TT0)−16.8VGrHGr−1.1VGrHGr(T⁄T0)+16HGr2VGr2

(11) fα(VGr,T)=0.794−16.8VGr2−0.0279(TT0)2+0.182(TT0)(1+VGr)

(12) fρ(VGr,T)=1.01−2.01VGr2−0.0131(TT0)

The graphene origami reinforcement may be dispersed in the copper matrix (Eqs. (13), (14))) with various distributions presented as follows:

(13) $$X-W_{Gr}:V_{Gr}\left(K\right)=2V_{Gr}\left|2K-N_{L}-1\right|/N_{L}$$

(14) $$U-W_{Gr}:{\;V}_{Gr}\left(K\right)=V_{Gr}$$

In which N_L_ is total number of layers.

The various distributions of folding (Eqs. (15), (16))) are depicted as follows:

(15) $$U-H_{Gr}:H_{Gr}\left(Z\right)=H_{Gr}$$

(16) $$X-H_{Gr}:H_{Gr}\left(Z\right)=H_{Gr}\;\cos\;\left(\frac{z}{h}\pi \right)$$

In which h is thickness and z is used for thickness coordinate.

**Fig. 1:**
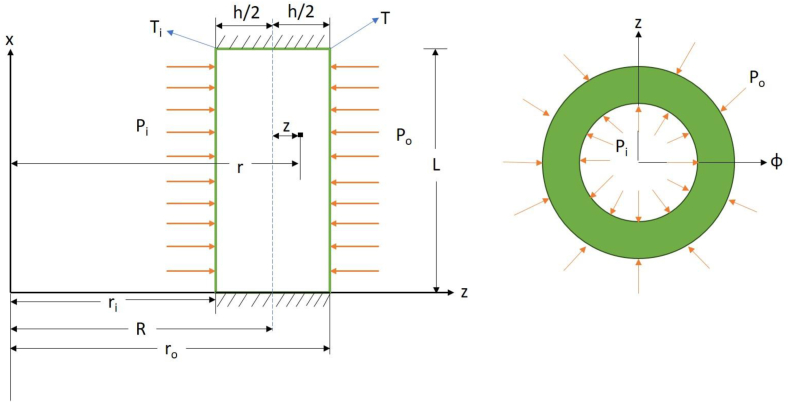
The schematic figure of a pressurized graphene origami reinforced cylindrical shell.

The governing equations are developed in continuation. The schematic figure of reinforced cylindrical shell is depicted in Fig. 1. The cylindrical shell is subjected to internal and external pressures with length L, thickness h and mean radius R. The coordinates) x, ϕ, z (are used for description of axial, circumferential and radial coordinates, respectively. The axial and radial displacements are denoted with U, W, respectively. Because of symmetric loading and material properties, the circumferential displacement is assumed zero.

The kinematic relations (Eqs. (17), (18))) are developed based on higher-order shear and normal deformation theory as follows [25]:

(17) $$u\left(x,\phi ,z\right)\approx u_{0}\left(x,\phi \right)+z\theta_{1}\left(x,\phi \right)+z^{2}u_{0}^{*}\left(x,\phi \right)+z^{3}\theta_{1}^{*}\left(x,\phi \right)$$

(18) $$w\left(x,\phi ,z\right)\approx w_{0}\left(x,\phi \right)+z\theta_{3}\left(x,\phi \right)+z^{2}w_{0}^{*}\left(x,\phi \right)+z^{3}\theta_{3}^{*}\left(x,\phi \right)$$

In which u, w are axial and radial displacements, u_0_, w_0_ are axial and radial displacements of middle surface, and θ_1_, u_0_*، w_0_*، θ_3_، θ_1_*, θ_3_* are lower and higher order rotations components. The strain components (Eqs. (19), (20), (21), (22)) using the proposed displacement field are derived as follows:

(19) $$\varepsilon_{x}=\left\{\varepsilon_{x}^{0}+z\kappa_{x}+z^{2}\varepsilon_{x}^{*}+z^{3}\kappa_{x}^{*}\right\}$$

(20) $$\varepsilon_{\phi }=\frac{1}{R}\left\{\varepsilon_{\phi }^{0}+z\kappa_{\phi }+z^{2}\varepsilon_{\phi }^{*}+z^{3}\kappa_{\phi }^{*}\right\}$$

(21) $$\varepsilon_{z}=\left\{\varepsilon_{z}^{0}+z\kappa_{z}+z^{2}\varepsilon_{z}^{*}+z^{3}\kappa_{z}^{*}\right\}$$

(22) $${ϒ}_{\text{xz}}=\left\{{ϒ}_{\text{xz}}^{0}+z\kappa_{\text{xz}}+z^{2}{ϒ}_{\text{xz}}^{*}+z^{3}\kappa_{\text{xz}}^{*}\right\}$$

In which the unknown variables (Eqs. (23), (24), (25), (26)) are obtained as follows:

(23) $$\left\{\varepsilon_{x}^{0}\;\kappa_{x}\;\varepsilon_{x}^{*}\;\kappa_{x}^{*}\right\}=\left\{\frac{\partial u_{0}}{\partial x}\;\frac{\partial \theta_{1}}{\partial x}\;\frac{\partial u_{0}^{*}}{\partial x}\;\frac{\partial \theta_{1}^{*}}{\partial x}\right\}$$

(24) $$\left\{\varepsilon_{\phi }^{0}\;\kappa_{\phi }\;\varepsilon_{\phi }^{*}\;\kappa_{\phi }^{*}\right\}=\left\{w_{0}\;\theta_{3}\;w_{0}^{*}\;\theta_{3}^{*}\right\}$$

(25) $$\left\{\varepsilon_{z}^{0}\;\kappa_{z}\;\varepsilon_{z}^{*}\;\kappa_{z}^{*}\right\}=\left\{\theta_{3}\;2w_{0}^{*}\;3\theta_{3}^{*}\;0\right\}$$

(26) $$\left\{{ϒ}_{xz}^{0}\;\kappa_{xz}\;{ϒ}_{xz}^{*}\;\kappa_{xz}^{*}\right\}=\left\{\frac{\partial w_{0}}{\partial x}+\theta_{1}\;\frac{\partial \theta_{3}}{\partial x}+2u_{0}^{*}\;\frac{\partial w_{0}^{*}}{\partial x}+3\theta_{1}^{*}\;\frac{\partial \theta_{3}^{*}}{\partial x}\right\}$$

The constitutive relations (Eq. (27)) are developed in presence of thermal loads as follows:

(27) $$\left[\begin{array}{c} \sigma_{x}\\ \sigma_{\phi }\\ \sigma_{z}\\ \tau_{x\phi }\\ \tau_{xz}\\ \tau_{\phi z} \end{array}\right]=\left[\begin{array}{cccccc} C_{11} & C_{12} & C_{13} & 0 & 0 & 0\\ C_{12} & C_{22} & C_{23} & 0 & 0 & 0\\ C_{13} & C_{23} & C_{33} & 0 & 0 & 0\\ 0 & 0 & 0 & C_{44} & 0 & 0\\ 0 & 0 & 0 & 0 & C_{55} & 0\\ 0 & 0 & 0 & 0 & 0 & C_{66} \end{array}\right]\left[\begin{array}{c} \varepsilon_{x}\\ \varepsilon_{\phi }\\ \varepsilon_{z}\\ \gamma_{x\phi }\\ \gamma_{xz}\\ \gamma_{\phi z} \end{array}\right]-\frac{\alpha_{c}E_{c}}{1-2\nu_{c}}\left[\begin{array}{c} \Delta T\\ \Delta T\\ \Delta T\\ 0\\ 0\\ 0 \end{array}\right]$$

In which the stiffness coefficients $C_{ij}$ (Eqs. (28), (29), (30)) are derived as follows:

(28) $$C_{11}=C_{22}=C_{33}=\frac{\left(1-\nu_{c}\right)E_{c}\left(z\right)}{\left(1+\nu_{c}\right)\left(1-2\nu_{c}\right)}$$

(29) $$C_{12}=C_{13}=C_{23}=\frac{\nu_{c}E_{c}\left(z\right)}{\left(1+\nu_{c}\right)\left(1-2\nu_{c}\right)}$$

(30) $$C_{44}=C_{55}=C_{66}=\frac{E_{c}\left(z\right)}{2\left(1+\nu_{c}\right)}$$

Principle of virtual work (Eq. (31)) yields to Ref. [39]:

(31) δU−δW=0

Or [40,41].

(32) δU=δW

in (Eq. (32)). The strain energy (Eq. (33)) for cylindrical shell is computed as follows:

(33) $$\delta U=\int_{-\frac{h}{2}}^{\frac{h}{2}}\int \left[\sigma_{x}\delta \varepsilon_{x}+\sigma_{\varphi }\delta \varepsilon_{\varphi }+\sigma_{z}\delta \varepsilon_{z}+\tau_{xz}\delta {ϒ}_{xz}\right]dAdz$$

The resultant components Nx، Mx، Nx*، Mx*، Qx، Qx*، Sx، Sx*، Nφ، Nφ*، Mφ Mφ*، A،, D (Eqs. (34), (35), (36)) are defined as follows:

(34) $$\left[\begin{array}{cc} \begin{array}{c} N_{x}\\ M_{x} \end{array} & \begin{array}{c} N_{\varphi }\\ M_{\varphi } \end{array}\\ \begin{array}{c} N_{x}^{*}\\ M_{x}^{*} \end{array} & \begin{array}{c} N_{\varphi }^{*}\\ M_{\varphi }^{*} \end{array} \end{array}\right]=\int_{z_{k-1}}^{z_{k}}\left[\begin{array}{c} \begin{array}{c} 1\\ z \end{array}\\ \begin{array}{c} z^{2}\\ z^{3} \end{array} \end{array}\right]\left[\begin{array}{cc} \sigma_{x} & \sigma_{\varphi } \end{array}\right]dz$$

(35) $$\left[\begin{array}{c} \begin{array}{c} Q_{x}\\ S_{x} \end{array}\\ \begin{array}{c} Q_{x}^{*}\\ S_{x}^{*} \end{array} \end{array}\right]=\int_{z_{k-1}}^{z_{k}}\left[\begin{array}{c} \begin{array}{c} 1\\ z \end{array}\\ \begin{array}{c} z^{2}\\ z^{3} \end{array} \end{array}\right]\left[\tau_{xz}\right]dz$$

(36) $$\left[\begin{array}{c} A\\ B\\ D \end{array}\right]=\int_{z_{k-1}}^{z_{k}}\left[\begin{array}{c} 1\\ z\\ z^{2} \end{array}\right]\left[\sigma_{z}\right]dz$$

The external work (Eqs. (37), (38))) is defined as follows:

(37) $$\delta W=\int_{0}^{2\pi }\int_{0}^{L}F\delta U_{z}dxd\phi$$

(38) $$F=\left[P_{i}\left(R-\frac{h}{2}\right)-P_{0}\left(R+\frac{h}{2}\right)\right]$$

Finally, the governing equations (Eqs. (39), (40), (41), (42), (43), (44), (45), (46)) are derived as follows:

(39) $$\delta u_{0}:\frac{\partial N_{x}}{\partial x}=0$$

(40) $$\delta w_{0}:\frac{\partial Q_{x}}{\partial x}-\frac{N_{\varphi }}{R}+F=0$$

(41) $$\delta \theta_{1}:\frac{\partial M_{x}}{\partial x}-Q_{x}=0$$

(42) $$\delta \theta_{3}:\frac{\partial S_{x}}{\partial x}-\frac{M_{\varphi }}{R}-A+FZ=0$$

(43) $$\delta u_{0}^{*}:\frac{\partial N_{x}^{*}}{\partial x}-2S_{x}=0$$

(44) $$\delta w_{0}^{*}:\frac{\partial Q_{x}^{*}}{\partial x}-\frac{N_{\varphi }^{*}}{R}-2B+FZ^{2}=0$$

(45) $$\delta \theta_{1}^{*}:\frac{\partial M_{x}^{*}}{\partial x}-3Q_{x}^{*}=0$$

(46) $$\delta \theta_{3}^{*}:\frac{\partial S_{x}^{*}}{\partial x}-\frac{M_{\varphi }^{*}}{R}-3D+FZ^{3}=0$$

in which (Eqs. (47), (48), (49)):

(47) $$\left\{A_{i}\right\}=\int \left\{z^{i-1}\right\}\frac{\left(1-\nu \right)E\left(z\right)}{\left(1+\nu \right)\left(1-2\nu \right)}dz,i=1\ldots 7$$

(48) $$\left\{B_{i}\right\}=\int \left\{z^{i-1}\right\}\frac{\nu E\left(z\right)}{\left(1+\nu \right)\left(1-2\nu \right)}dz,i=1\ldots 7$$

(49) $$\left\{D_{i}\right\}=\int \left\{z^{i-1}\right\}\frac{E\left(z\right)}{2\left(1+\nu \right)}dz,i=1\ldots 7$$

## 3 Solution

The analytical solution is derived in this section. The static results can be obtained using Eigenvalue-Eigenvector method for clamped-clamped boundary conditions. The general solution (Eqs. (50), (51))) is included homogeneous ${\left\{y\right\}}_{h}$ and particular ${\left\{y\right\}}_{p}$ solutions as follows:

(50) $$\left\{y\right\}={\left\{y\right\}}_{h}+{\left\{y\right\}}_{p}$$

(51) $$\left[G_{1}\right]\frac{d^{2}}{dx^{2}}\left\{y\right\}+\left[G_{2}\right]\frac{d}{dx}\left\{y\right\}+\left[G_{3}\right]\left\{y\right\}=\left\{N\right\}$$

By assuming the homogeneous solution as ${\left\{y\right\}}_{h}=\left\{ℵ\right\}e^{mx}$, the homogeneous solution is obtained. In homogeneous solution, $m_{i}$ are roots of characteristic equation (Eq. (52)) and ${ℵ}_{i}$ are eigenvectors that defined as:

(52) $$\left[G_{1}\right]m^{2}+\left[G_{2}\right]m+\left[G_{3}\right]=0$$

In general state, the homogeneous solution (Eq. (53)) is defined as:

(53) $${\left\{y\right\}}_{h}=\sum_{1}^{16}c_{i}{\left\{ℵ\right\}}_{i}e^{m_{i}x}$$

In which ${\left\{ℵ\right\}}_{i}$ are eigenvector components. The particular solution (Eq. (54)) is obtained using the last term of left hand of Eq. (51) as follows:

(54) $$\left\{y\right\}={\left[G_{3}\right]}^{-1}\left\{N\right\}$$

The numerical solution is obtained with applying the clamped-clamped boundary conditions (Eq. (55)) as follows:

(55) $$B.C:\left\{\begin{array}{c} \begin{array}{c} \begin{array}{c} u_{0}=0\\ \theta_{1}=0 \end{array}\\ \begin{array}{c} u_{0}^{*}=0\\ \theta_{1}^{*}=0 \end{array} \end{array}\\ \begin{array}{c} \begin{array}{c} w_{0}=0\\ \theta_{3}=0 \end{array}\\ \begin{array}{c} w_{0}^{*}=0\\ \theta_{3}^{*}=0 \end{array} \end{array} \end{array}\right\}\;at\;x=0,l$$

## 4 Numerical results and discussion

In this section, the numerical results and corresponding discussion are presented. The results are included radial and axial displacement, radial, axial and circumferential stress and strain components along the axial and radial direction in terms of volume fraction and folding degree of graphene origami, various distributions and geometric parameters [97].

Before presentation of the numerical results, the material properties should be presented. Listed in Table 1, Table 2 are material and geometric characteristics of graphene origami reinforced copper matrix and cylindrical shell, respectively [37,94,96,95].

**Table 1:** The material properties of graphene origami and copper matrix.

l_Gr_	83.76 × 10^−10^ m	E_Gr_	929.57 GPa
t_Gr_	3.4 × 10^−10^ m	E_Cu_	65.79 GPa
ρ_Gr_	1.8 g/cm^3^	ρ_Cu_	8.8 g/cm^3^
ν_Gr_	0.22	ν_Cu_	0.387

**Table 2:** The geometric and loading characteristics of cylindrical shell.

L	1.5 m	P_0_	0.1 MPa
R	0.3 L	P_i_	80 MPa
h	0.25 R	T_0_	300 K

**Fig. 2:**
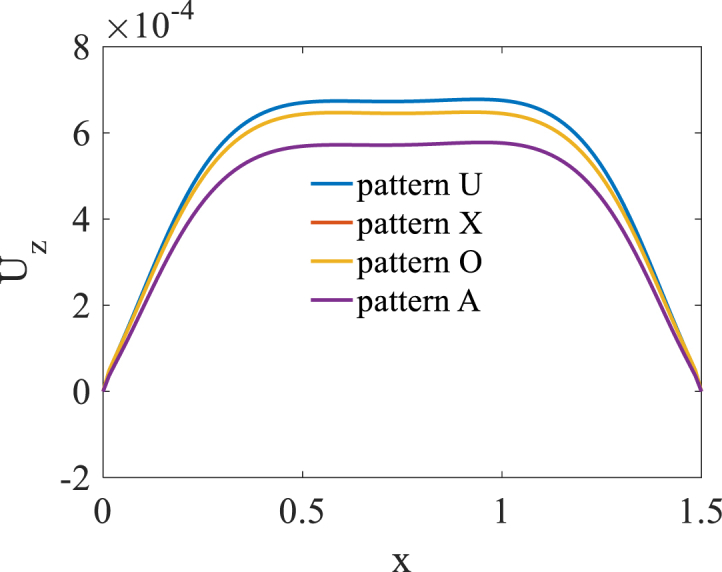
Longitudinal variation of radial displacement $U_{z}$ forU-W_Gr_, X-W_Gr_distributions.

**Fig. 3:**
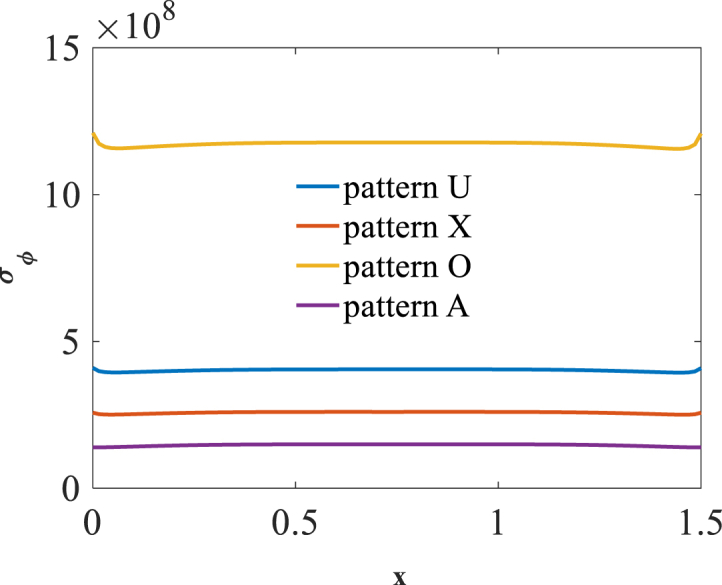
Longitudinal variation of circumferential stress $\sigma_{\phi }$ forU-W_Gr_, X-W_Gr_distributions.

**Fig. 4:**
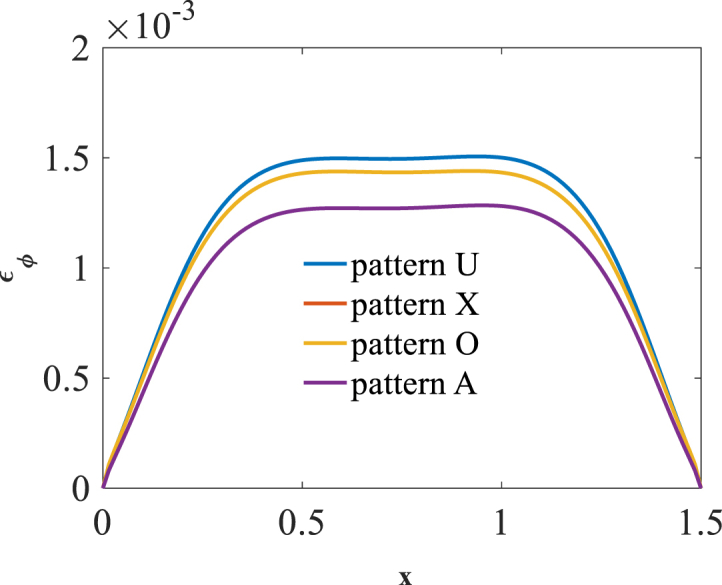
Longitudinal variation of circumferential strain $\varepsilon_{\phi }$ forU-W_Gr_, X-W_Gr_distributions.

Fig. 2, 3 and 4 present longitudinal variation of radial displacement $U_{z}$, circumferential stress $\sigma_{\phi }$ and strain $\varepsilon_{\phi }$ for two U-pattern and X-pattern distributions of graphene origami. Because of more stiffness of X-pattern distribution of graphene origami, a lower deflection for this distribution is observed. Regard to the circumferential strain and stress components, it is observed that the maximum ones are occurred for U-pattern and X-pattern distributions of graphene origami, respectively.

**Fig. 5:**
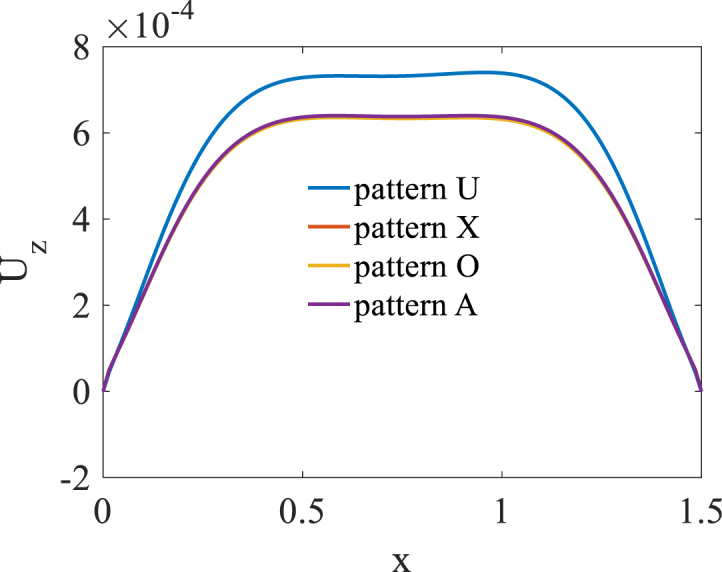
Longitudinal variation of radial displacement $U_{z}$ for U-H_Gr_ and X-H_Gr_ distributions.

**Fig. 6:**
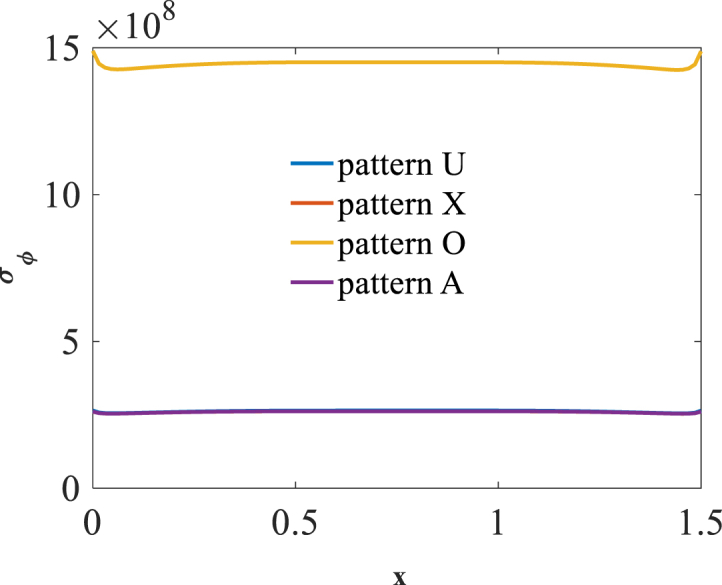
Longitudinal variation of circumferential stress $\sigma_{\phi }$ for U-H_Gr_ and X-H_Gr_ distributions.

**Fig. 7:**
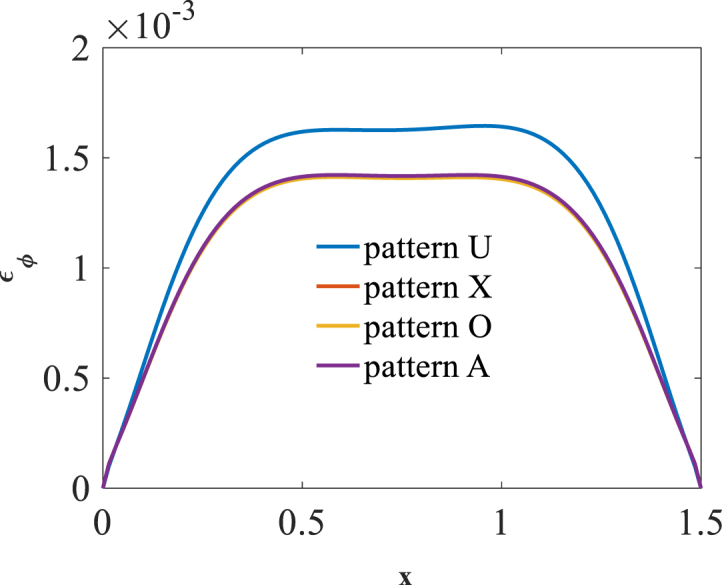
Longitudinal variation of circumferential strain $\varepsilon_{\phi }$ for U-H_Gr_ and X-H_Gr_ distributions.

Shown in Fig. 5, Fig. 6, Fig. 7 are longitudinal variation of radial displacement $U_{z}$, circumferential stress $\sigma_{\phi }$ and strain $\varepsilon_{\phi }$ for two U-H_Gr_ and X-H_Gr_ distributions of folding degree. One can conclude that the deflection and consequently the circumferential strain for U-H_Gr_ is more than X-H_Gr_ because of lower stiffness. Furthermore, the circumferential stress for X-H_Gr_ is more than U-H_Gr_ because of more modulus of elasticity and stiffness. The results of these figures are obtained for the following characteristics:

$$H_{\text{Gr}}=100\%،\;W_{\text{Gr}}=0.5\%،T=400$$

**Fig. 8:**
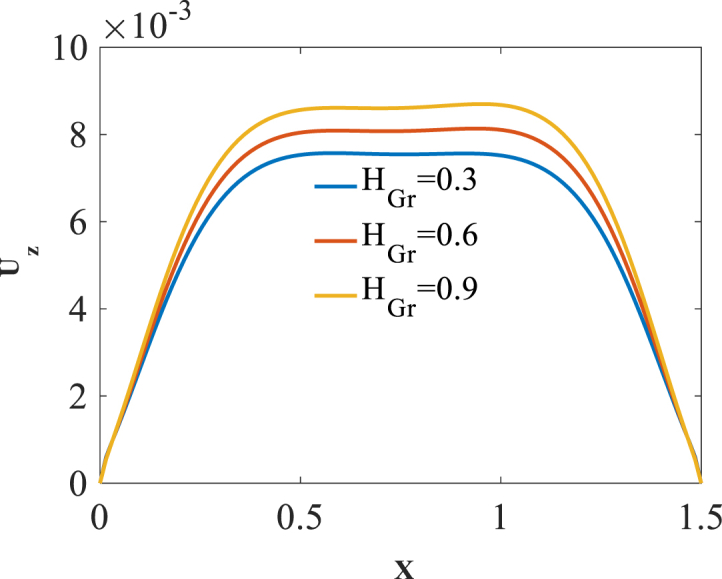
Longitudinal variation of $U_{z}$ with changes of folding degrees H_Gr_.

**Fig. 9:**
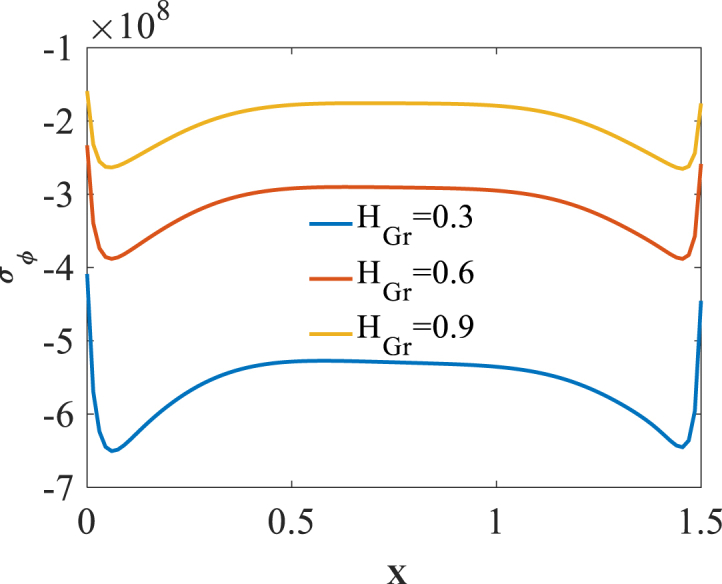
Longitudinal variation of $\sigma_{\phi }$ with changes of folding degrees H_Gr_.

**Fig. 10:**
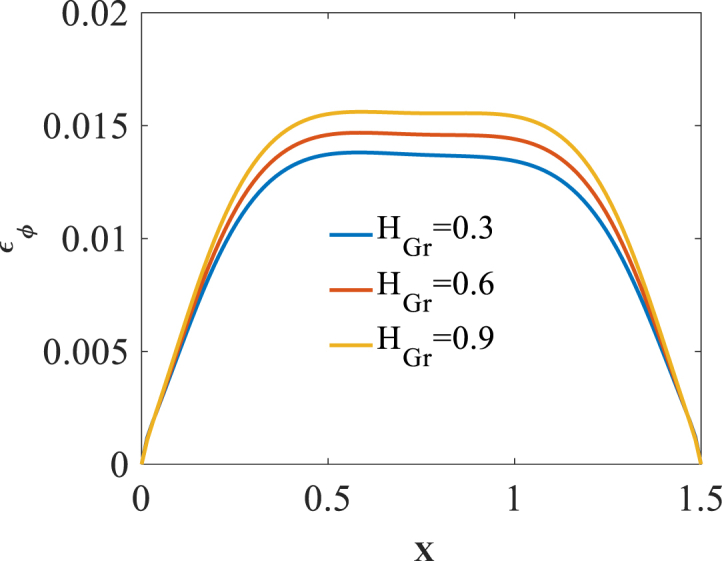
Longitudinal variation of $\varepsilon_{\phi }$ with changes of folding degrees H_Gr_.

To investigate effect of folding degree H_Gr_ on the bending responses, Fig. 8, Fig. 9, Fig. 10 are produced. Presented in Fig. 8, Fig. 9, Fig. 10 are longitudinal variation of radial displacement $U_{z}$, circumferential stress $\sigma_{\phi }$ and strain $\varepsilon_{\phi }$ with changes of folding degrees H_Gr_. One can find a significant increase in radial displacement $U_{z}$ and circumferential strain $\varepsilon_{\phi }$ because of a decrease in structural stiffness with an increase in folding degree H_Gr_. Investigating the changes of circumferential stress $\sigma_{\phi }$ reflects a decrease in absolute value of this component with an increase in folding degrees H_Gr_. The latest change is because of a decrease in structural stiffness with an increase in folding degrees H_Gr_. The results of these figures are obtained for the following characteristics:

$$W_{\text{Gr}}=0.5\%،T=400k$$

**Fig. 11:**
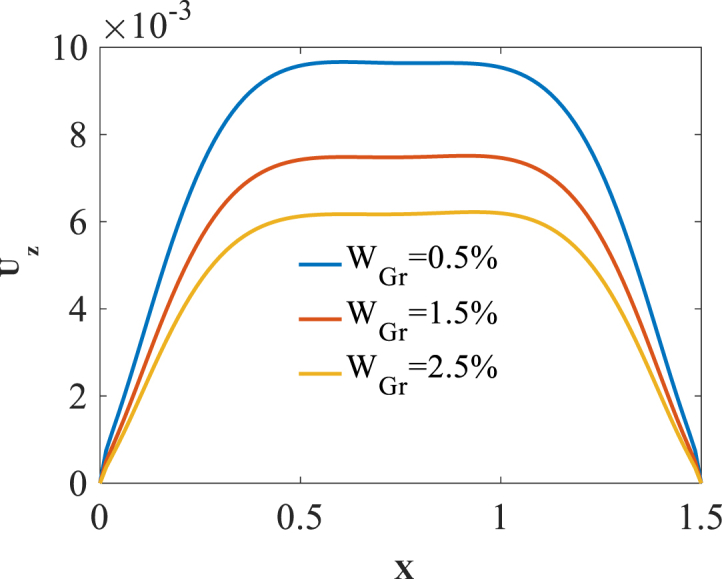
Longitudinal variation of $U_{z}$ with changes of volume fraction W_Gr_.

**Fig. 12:**
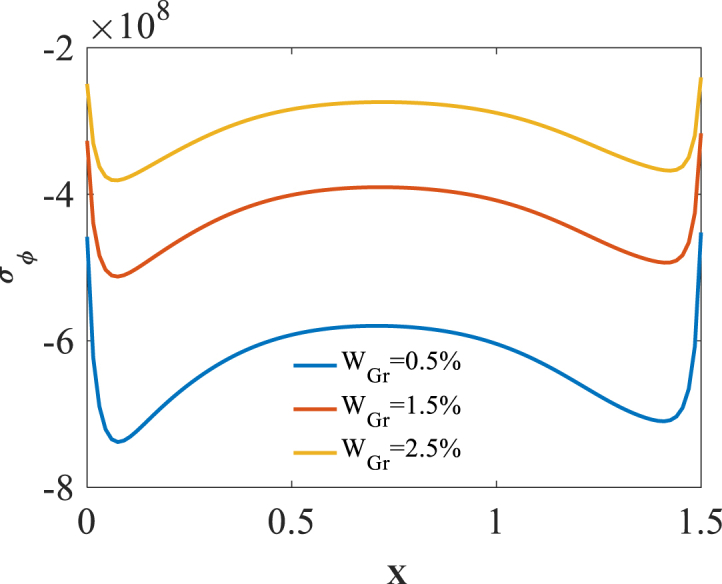
Longitudinal variation of $\sigma_{\phi }$ with changes of volume fraction W_Gr_.

**Fig. 13:**
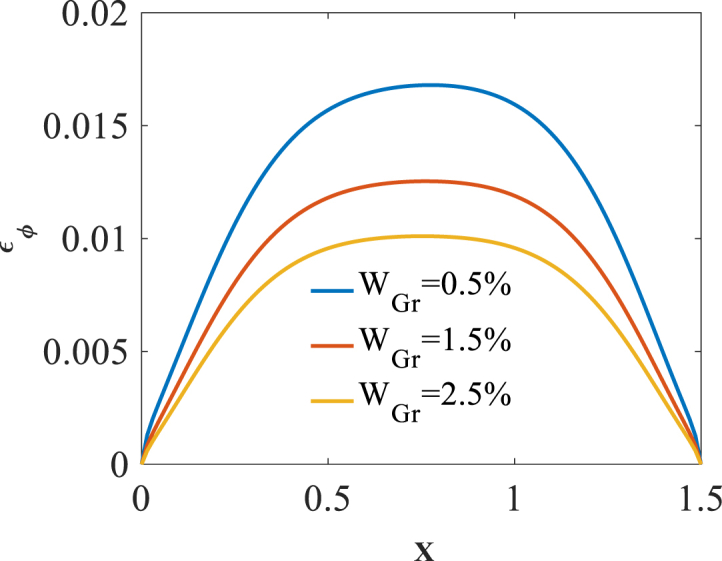
Longitudinal variation of circumferential strain $\varepsilon_{\phi }$ with changes of volume fraction W_Gr_.

To investigate effect of volume fraction W_Gr_ on the bending responses, Fig. 11, Fig. 12, Fig. 13 are produced. Presented in Fig. 11, Fig. 12, Fig. 13 are longitudinal variation of radial displacement $U_{z}$, circumferential stress $\sigma_{\phi }$ and strain $\varepsilon_{\phi }$ with changes of volume fraction W_Gr_. One can find a significant decrease in radial displacement $U_{z}$, circumferential stress $\sigma_{\phi }$ and strain $\varepsilon_{\phi }$ because of an increase in structural stiffness with an increase in volume fraction W_Gr_. The results of these figures are obtained for the following characteristics:

$$H_{\text{Gr}}=100\%،T=400k$$

**Fig. 14:**
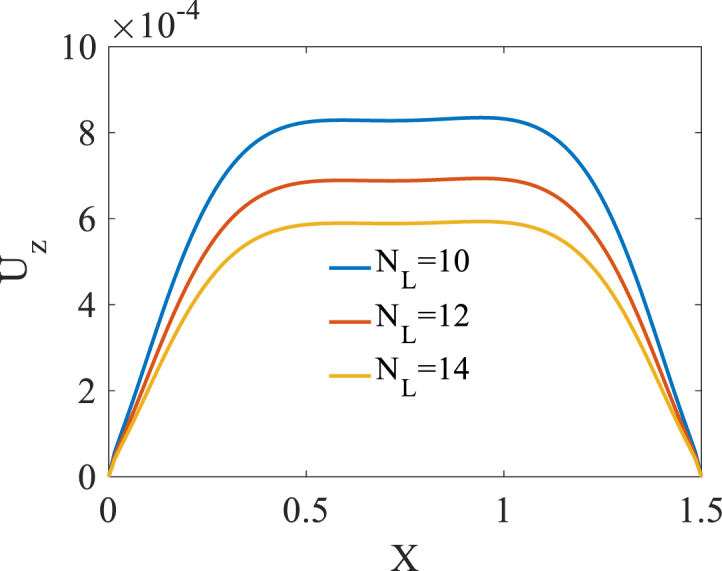
Longitudinal variation of radial displacement $U_{z}$ with changes of number of layers $N_{L}$.

**Fig. 15:**
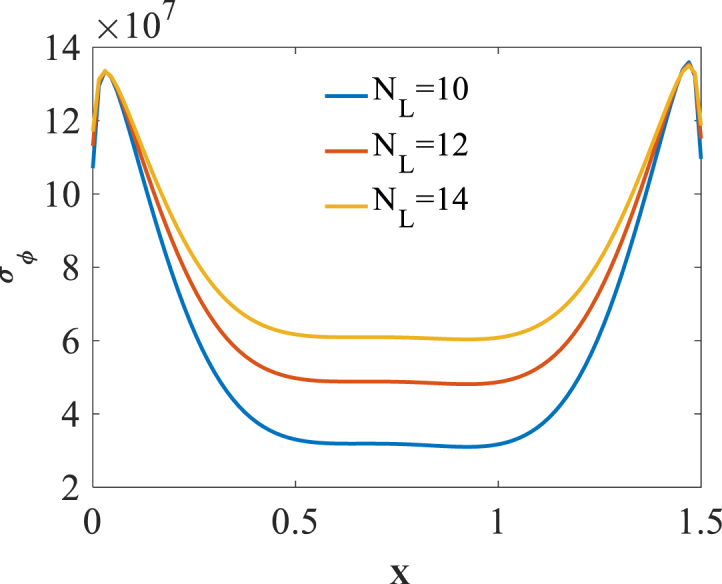
Longitudinal variation of circumferential stress $\sigma_{\phi }$ with changes of number of layers $N_{L}$.

**Fig. 16:**
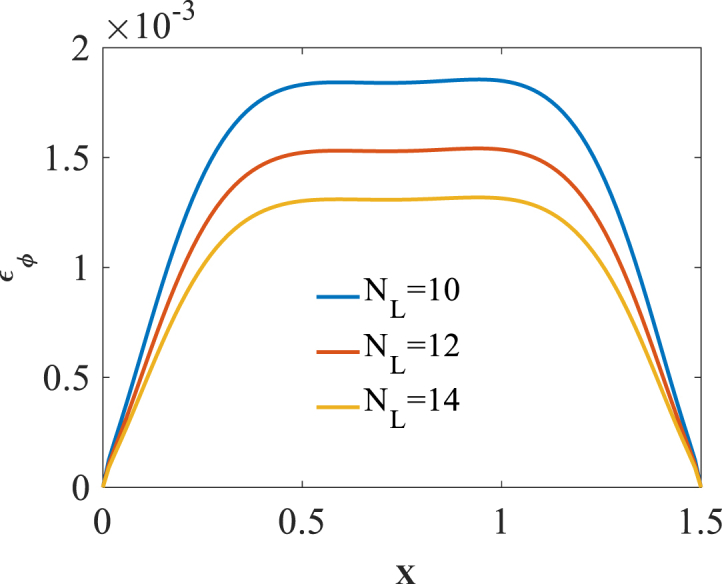
Longitudinal variation of circumferential strain $\varepsilon_{\phi }$ with changes of number of layers $N_{L}$.

The effect of number of layers $N_{L}$ is studied on the bending responses of graphene origami reinforced cylindrical pressure vessel. Presented in Fig. 14, Fig. 15, Fig. 16 are longitudinal variation of radial displacement $U_{z}$, circumferential stress $\sigma_{\phi }$ and strain $\varepsilon_{\phi }$ with changes of number of layers $N_{L}$. A significant decrease in radial displacement $U_{z}$, and circumferential strain $\varepsilon_{\phi }$ is observed with an increase in number of layers $N_{L}$ because of an increase in structural stiffness of graphene origami reinforced cylindrical shell. In addition, circumferential stress $\sigma_{\phi }$ is increased with an increase in number of layers $N_{L}$. The results of these figures are obtained for the following characteristics:

$$H_{\text{Gr}}=100\%،T=400k,W_{\text{Gr}}=0.5\%$$

**Fig. 17:**
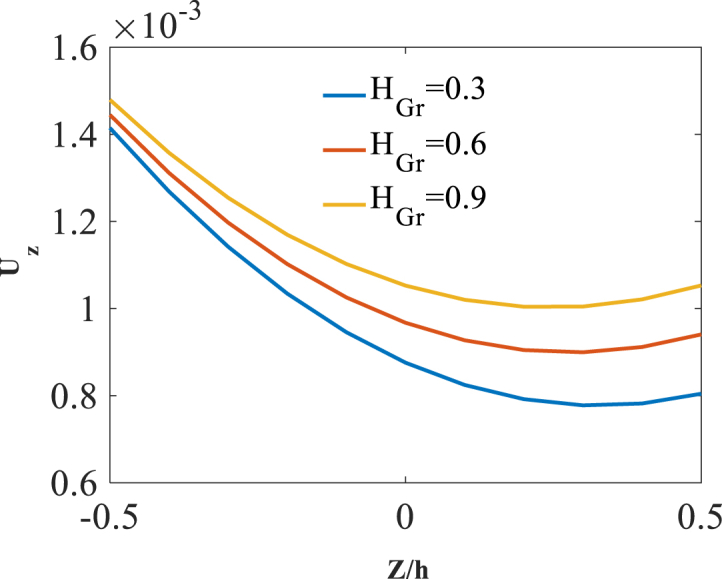
Radial variation of radial displacement $U_{z}$ with changes of folding degree H_Gr_.

**Fig. 18:**
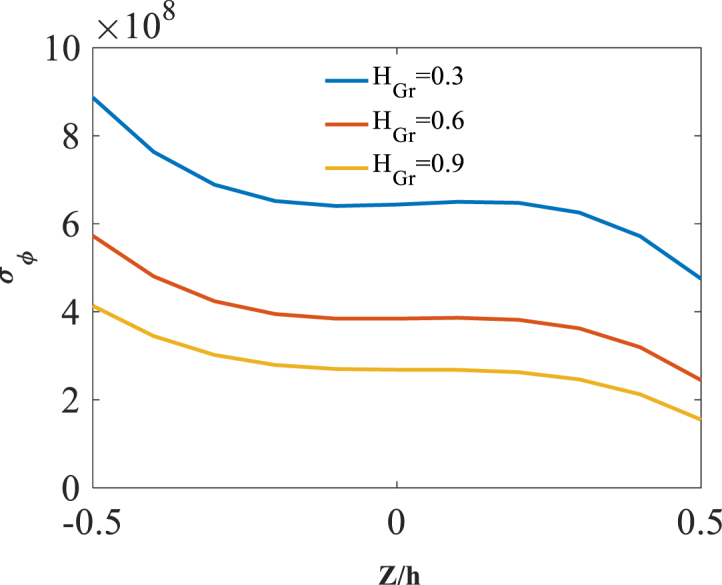
Radial variation of circumferential stress $\sigma_{\phi }$ with changes of folding degree H_Gr_.

**Fig. 19:**
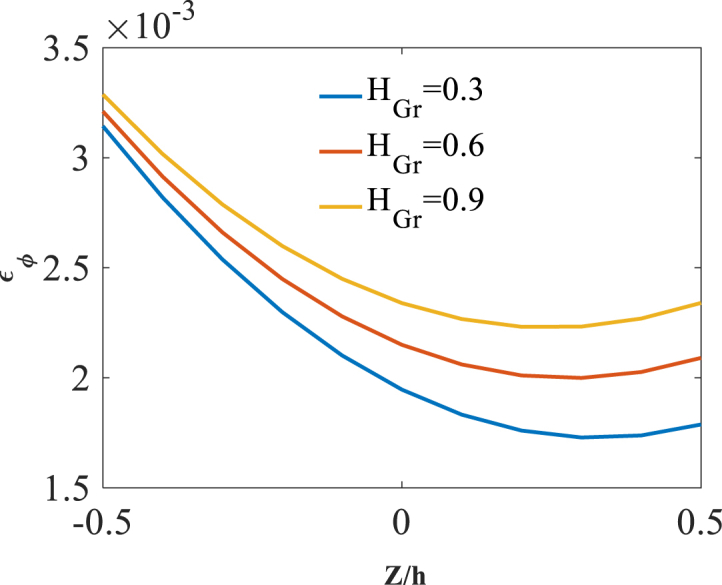
Radial variation of circumferential strain $\varepsilon_{\phi }$ with changes of folding degree H_Gr_.

As last parameter, the effect of radial coordinate is investigated through presentation of results along the thickness direction z. Shown in Fig. 17, Fig. 18, Fig. 19 are radial variation of radial displacement $U_{z}$, circumferential stress $\sigma_{\phi }$ and strain $\varepsilon_{\phi }$ with changes of folding degree H_Gr_. A significant increase in radial displacement $U_{z}$, circumferential stress $\sigma_{\phi }$ and strain $\varepsilon_{\phi }$ is observed with an increase in folding degree H_Gr_ because of a decrease in structural stiffness of graphene origami reinforced cylindrical shell. The results of these figures are obtained for the following characteristics:

T=400kوW_(Gr)=0.5%

## 5 Conclusion

In this paper, the bending analysis of a functionally graded graphene origami reinforced cylindrical shell subjected to internal/external pressures was presented. Higher-order shear and normal deformation theory was extended to derive kinematic relations in axisymmetric case. The constitutive relations were extended for the composite cylindrical shell. Some modifier coefficients were employed for correction of the mentioned material properties in terms of volume fraction and folding degree of graphene origami, characteristics of copper and graphene nanoplatelets and thermal loads. The principle of virtual work was used to derive governing equations. The static bending results including radial and axial displacements, circumferential strain and stress were evaluated along the longitudinal and radial directions in terms of volume fraction and folding degree of graphene origami and its distribution. The main results of this study is expressed as follows:

Investigating effect of various distribution of graphene origami shows the higher value of displacement and stress for uniform distribution because of lower bending stiffness.

Various distributions of folding degree lead to this output that the stress for X-pattern is more than uniform one while deformation of uniform distribution is dominant.

An enhancement in volume fraction of graphene origami leads to more stiffness and consequently a decrease in deformation, stress and strain components.

Investigating effect of number of layers on the bending responses indicates that the deformation, strain and stress components are decreased with an increase in this parameter because of an increase in structural stiffness.

## Data availability statement

No data was used for the research described in the article.

## CRediT authorship contribution statement

**Mohammad Hossein Samadzadeh:** Software, Resources, Project administration, Methodology, Investigation, Data curation. **Mohammad Arefi:** Writing – review & editing, Writing – original draft, Supervision, Methodology, Formal analysis, Conceptualization. **Abbas Loghman:** Writing – original draft, Supervision, Formal analysis, Conceptualization.

## Declaration of competing interest

The authors declare that they have no known competing financial interests or personal relationships that could have appeared to influence the work reported in this article.

## Appendix

$$N_{x}=\int \sigma_{x}dz=A_{1}\frac{\partial u_{0}}{\partial x}+A_{2}\frac{\partial \theta_{1}}{\partial x}+A_{3}\frac{\partial u_{0}^{*}}{\partial x}+A_{4}\frac{\partial \theta_{1}^{*}}{\partial x}+\frac{1}{R}\left(B_{1}w_{0}+B_{2}\theta_{3}+B_{3}w_{0}^{*}+B_{4}\theta_{3}^{*}\right)+B_{1}\theta_{3}+B_{2}\left(2w_{0}^{*}\right)+B_{3}\left(3\theta_{3}^{*}\right)$$

$$M_{x}=\int z\sigma_{x}dz=A_{2}\frac{\partial u_{0}}{\partial x}+A_{3}\frac{\partial \theta_{1}}{\partial x}+A_{4}\frac{\partial u_{0}^{*}}{\partial x}+A_{5}\frac{\partial \theta_{1}^{*}}{\partial x}+\frac{1}{R}\left(B_{2}w_{0}+B_{3}\theta_{3}+B_{4}w_{0}^{*}+B_{5}\theta_{3}^{*}\right)+B_{2}\theta_{3}+B_{3}\left(2w_{0}^{*}\right)+B_{4}\left(3\theta_{3}^{*}\right)$$

$$N_{x}^{*}=\int {z^{2}\sigma }_{x}dz=A_{3}\frac{\partial u_{0}}{\partial x}+A_{4}\frac{\partial \theta_{1}}{\partial x}+A_{5}\frac{\partial u_{0}^{*}}{\partial x}+A_{6}\frac{\partial \theta_{1}^{*}}{\partial x}+\frac{1}{R}\left(B_{3}w_{0}+B_{4}\theta_{3}+B_{5}w_{0}^{*}+B_{6}\theta_{3}^{*}\right)+B_{3}\theta_{3}+B_{4}\left(2w_{0}^{*}\right)+B_{5}\left(3\theta_{3}^{*}\right)$$

$$M_{x}^{*}=\int z^{3}\sigma_{x}dz=A_{4}\frac{\partial u_{0}}{\partial x}+A_{5}\frac{\partial \theta_{1}}{\partial x}+A_{6}\frac{\partial u_{0}^{*}}{\partial x}+A_{7}\frac{\partial \theta_{1}^{*}}{\partial x}+\frac{1}{R}\left(B_{4}w_{0}+B_{5}\theta_{3}+B_{6}w_{0}^{*}+B_{7}\theta_{3}^{*}\right)+B_{4}\theta_{3}+B_{5}\left(2w_{0}^{*}\right)+B_{6}\left(3\theta_{3}^{*}\right)$$

$$N_{\varphi }=\int \sigma_{\varphi }dz=\frac{1}{R}\left(A_{1}w_{0}+A_{2}\theta_{3}+A_{3}w_{0}^{*}+A_{4}\theta_{3}^{*}\right)+B_{1}\frac{\partial u_{0}}{\partial x}+B_{2}\frac{\partial \theta_{1}}{\partial x}+B_{3}\frac{\partial u_{0}^{*}}{\partial x}+B_{4}\frac{\partial \theta_{1}^{*}}{\partial x}+B_{1}\theta_{3}+B_{2}\left(2w_{0}^{*}\right)+B_{3}\left(3\theta_{3}^{*}\right)$$

$$M_{\varphi }=\int z\sigma_{\varphi }dz=\frac{1}{R}\left(A_{2}w_{0}+A_{3}\theta_{3}+A_{4}w_{0}^{*}+A_{5}\theta_{3}^{*}\right)+B_{2}\frac{\partial u_{0}}{\partial x}+B_{3}\frac{\partial \theta_{1}}{\partial x}+B_{4}\frac{\partial u_{0}^{*}}{\partial x}+B_{5}\frac{\partial \theta_{1}^{*}}{\partial x}+B_{2}\theta_{3}+B_{3}\left(2w_{0}^{*}\right)+B_{4}\left(3\theta_{3}^{*}\right)$$

$$N_{\varphi }^{*}=\int {z^{2}\sigma }_{\varphi }dz=\frac{1}{R}\left(A_{3}w_{0}+A_{4}\theta_{3}+A_{5}w_{0}^{*}+A_{6}\theta_{3}^{*}\right)+B_{3}\frac{\partial u_{0}}{\partial x}+B_{4}\frac{\partial \theta_{1}}{\partial x}+B_{5}\frac{\partial u_{0}^{*}}{\partial x}+B_{6}\frac{\partial \theta_{1}^{*}}{\partial x}+B_{3}\theta_{3}+B_{4}\left(2w_{0}^{*}\right)+B_{5}\left(3\theta_{3}^{*}\right)$$

$$M_{\varphi }^{*}=\int {z^{3}\sigma }_{\varphi }dz=\frac{1}{R}\left(A_{4}w_{0}+A_{5}\theta_{3}+A_{6}w_{0}^{*}+A_{7}\theta_{3}^{*}\right)+B_{4}\frac{\partial u_{0}}{\partial x}+B_{5}\frac{\partial \theta_{1}}{\partial x}+B_{6}\frac{\partial u_{0}^{*}}{\partial x}+B_{7}\frac{\partial \theta_{1}^{*}}{\partial x}+B_{4}\theta_{3}+B_{5}\left(2w_{0}^{*}\right)+B_{6}\left(3\theta_{3}^{*}\right)$$

$$Q_{x}=\int \tau_{xz}dz=C_{1}\left(\frac{\partial w_{0}}{\partial x}+\theta_{1}\right)+C_{2}\left(\frac{\partial \theta_{3}}{\partial x}+2u_{0}^{*}\right)+C_{3}\left(\frac{\partial w_{0}^{*}}{\partial x}+3\theta_{1}^{*}\right)+C_{4}\left(\frac{\partial \theta_{3}^{*}}{\partial x}\right)$$

$$S_{x}=\int z\tau_{xz}dz=C_{2}\left(\frac{\partial w_{0}}{\partial x}+\theta_{1}\right)+C_{3}\left(\frac{\partial \theta_{3}}{\partial x}+2u_{0}^{*}\right)+C_{4}\left(\frac{\partial w_{0}^{*}}{\partial x}+3\theta_{1}^{*}\right)+C_{5}\left(\frac{\partial \theta_{3}^{*}}{\partial x}\right)$$

$$Q_{x}^{*}=\int {z^{2}\tau }_{xz}dz=C_{3}\left(\frac{\partial w_{0}}{\partial x}+\theta_{1}\right)+C_{4}\left(\frac{\partial \theta_{3}}{\partial x}+2u_{0}^{*}\right)+C_{5}\left(\frac{\partial w_{0}^{*}}{\partial x}+3\theta_{1}^{*}\right)+C_{6}\left(\frac{\partial \theta_{3}^{*}}{\partial x}\right)$$

$$S_{x}^{*}=\int z^{3}\tau_{xz}dz=C_{4}\left(\frac{\partial w_{0}}{\partial x}+\theta_{1}\right)+C_{5}\left(\frac{\partial \theta_{3}}{\partial x}+2u_{0}^{*}\right)+C_{6}\left(\frac{\partial w_{0}^{*}}{\partial x}+3\theta_{1}^{*}\right)+C_{7}\left(\frac{\partial \theta_{3}^{*}}{\partial x}\right)$$

$$A=\int \sigma_{z}dz=A_{1}\theta_{3}+A_{2}\left(2w_{0}^{*}\right)+A_{3}\left(3\theta_{3}^{*}\right)+\frac{1}{R}\left(B_{1}w_{0}+B_{2}\theta_{3}+B_{3}w_{0}^{*}+B_{4}\theta_{3}^{*}\right)+B_{1}\frac{\partial u_{0}}{\partial x}+B_{2}\frac{\partial \theta_{1}}{\partial x}+B_{3}\frac{\partial u_{0}^{*}}{\partial x}+B_{4}\frac{\partial \theta_{1}^{*}}{\partial x}$$

$$B=\int z\sigma_{z}dz=A_{2}\theta_{3}+A_{3}\left(2w_{0}^{*}\right)+A_{4}\left(3\theta_{3}^{*}\right)+\frac{1}{R}\left(B_{2}w_{0}+B_{3}\theta_{3}+B_{4}w_{0}^{*}+B_{5}\theta_{3}^{*}\right)+B_{2}\frac{\partial u_{0}}{\partial x}+B_{3}\frac{\partial \theta_{1}}{\partial x}+B_{4}\frac{\partial u_{0}^{*}}{\partial x}+B_{5}\frac{\partial \theta_{1}^{*}}{\partial x}$$

$$D=\int z^{2}\sigma_{z}dz=A_{3}\theta_{3}+A_{4}\left(2w_{0}^{*}\right)+A_{5}\left(3\theta_{3}^{*}\right)+\frac{1}{R}\left(B_{3}w_{0}+B_{4}\theta_{3}+B_{5}w_{0}^{*}+B_{6}\theta_{3}^{*}\right)+B_{3}\frac{\partial u_{0}}{\partial x}+B_{4}\frac{\partial \theta_{1}}{\partial x}+B_{5}\frac{\partial u_{0}^{*}}{\partial x}+B_{6}\frac{\partial \theta_{1}^{*}}{\partial x}$$
